# Eye Movements in Real-World Scene Photographs: General Characteristics and Effects of Viewing Task

**DOI:** 10.3389/fpsyg.2019.02915

**Published:** 2020-01-14

**Authors:** Deborah A. Cronin, Elizabeth H. Hall, Jessica E. Goold, Taylor R. Hayes, John M. Henderson

**Affiliations:** ^1^Center for Mind and Brain, University of California, Davis, Davis, CA, United States; ^2^Department of Psychology, University of California, Davis, Davis, CA, United States

**Keywords:** eye movements, scene perception, task instruction, gaze control, saccades

## Abstract

The present study examines eye movement behavior in real-world scenes with a large (*N* = 100) sample. We report baseline measures of eye movement behavior in our sample, including mean fixation duration, saccade amplitude, and initial saccade latency. We also characterize how eye movement behaviors change over the course of a 12 s trial. These baseline measures will be of use to future work studying eye movement behavior in scenes in a variety of literatures. We also examine effects of viewing task on when and where the eyes move in real-world scenes: participants engaged in a memorization and an aesthetic judgment task while viewing 100 scenes. While we find no difference at the mean-level between the two tasks, temporal- and distribution-level analyses reveal significant task-driven differences in eye movement behavior.

## Introduction

Due to the acuity limits of peripheral vision, we must move our eyes to explore the world’s rich detail. With each fixation, a new region of the world is brought into focus. The duration of each fixation and the amplitude of the saccades between them vary with the contents of the current scene, the viewer’s task, and unique aspects of the individual viewer. Where the eyes move in a given scene is similarly variable. In the present study, we explore eye movement behaviors in detail with a large sample.

A wide range of disciplines study eye movements in photographs of scenes. Cognitive psychologists use these measures to study perception (e.g., Currie et al., 2000; Gajewski and Henderson, 2005; Henderson and Hollingworth, 1999; Rayner et al., 2009; Smith et al., 2012), attention (e.g., Brockmole and Henderson, 2005a, b; Brockmole and Võ, 2010; Wolfe et al., 2011a, b; Henderson and Hayes, 2017, 2018; Peacock et al., 2019) memory processes (e.g., Irwin and Zelinsky, 2002; Castelhano and Henderson, 2005; Hannula et al., 2012; Võ and Wolfe, 2012, 2013; Olejarczyk et al., 2014; Ramey et al., 2019), and language (e.g., Henderson and Ferreira, 2004; Altmann and Kamide, 2007, 2009; Henderson et al., 2018), among other topics. Measures of eye movements in scenes are used in social psychology (Chua et al., 2005; Birmingham et al., 2008; Risko et al., 2012), clinical psychology (Fletcher-Watson et al., 2009; Hayes and Henderson, 2018; Tseng et al., 2013), and developmental psychology (Açik et al., 2010; Koski et al., 2013; Amso et al., 2014; Helo et al., 2014; van Renswoude et al., 2019). Human eye movements in scenes are also used to validate models of computer vision in engineering and computer science applications (Ehinger et al., 2009; Subramanian et al., 2011; Bylinskii et al., 2016). A notable example of the usefulness of human eye movement data to computer science and modeling applications comes from the MIT Saliency Benchmark, a project that has spurred a large amount of research on fixation behaviors in scenes (Judd et al., 2012; Borji et al., 2013; Borji and Itti, 2015; Bylinskii et al., 2016).

While the general temporal characteristics of eye movements and fixations during reading is well described (Rayner, 2009; Clifton et al., 2016), eye movement behavior in real-world scenes is less well characterized despite its widespread use. Typical saccade amplitudes and fixation durations have been described by relatively small-N studies and reviews over the years. From these studies, it is thought that saccades in scenes tend to be 2–4° in amplitude (Henderson and Hollingworth, 1998) and fixations tend to last 200–300 ms (Rayner, 2009). To our knowledge, there has not been a large-N examination of the temporal properties of saccades and fixations in scenes. Establishing baseline metrics of eye movement behaviors in scenes is important to both experimental and clinical research as it allows for the identification of “typical” and “atypical” patterns of attention. Therefore, a goal of the present study was to characterize eye movement behavior in scenes with viewing data from 100 participants.

General estimates of saccade amplitude and fixation durations are valuable to our understanding of how we process scenes. However, both saccade amplitude and fixation duration are influenced by a variety of factors. Fixations tend to last longer on regions of a scene that are more complex (e.g., high edge-density, clutter, etc.; Henderson, 2003; Rayner, 2009; Nuthmann, 2017) or less discriminable (e.g., lower in luminance, low-pass filtered, etc.; Loftus, 1985; Parkhust et al., 2000). Under some conditions, low-level features of a scene are postulated to draw the eyes, thereby influencing the size of saccades (Koch and Ullman, 1985; Mannan et al., 1996, 1997; Itti and Koch, 2000, 2001; Parkhurst et al., 2002). While these low-level features can influence eye movement behavior, the bulk of natural viewing behavior serves cognitive processes in a top-down fashion. Eye movements are guided to the most meaningful or informative regions of a scene rather than the most visually salient regions (e.g., Torralba, 2003; Rothkopf et al., 2007; Henderson and Hayes, 2017, 2018; Peacock et al., 2019) and linger there for longer (e.g., Loftus and Mackworth, 1978; Friedman, 1979; Henderson et al., 1999; Võ and Henderson, 2011). This top-down guidance of eye movements is driven by factors including the viewer’s knowledge, short- and long-term memory, and task goals (for a review, see Henderson, 2011).

During every-day tasks, the eyes move almost exclusively to the most relevant objects and regions for the task at hand. A multitude of studies tracking the eyes during sandwich- and tea-making (Land et al., 1999; Land and Hayhoe, 2001), driving (Land and Lee, 1994; Land and Tatler, 2001; Chattington et al., 2007), walking (Jovancevic and Hayhoe, 2009), and athletic activity (Land and McLeod, 2000; Hayhoe et al., 2005; Hagemann et al., 2010; for a review of eye movements in every-day tasks, see Land, 2006) find evidence of top-down, task-directed eye movements rather than bottom-up movements of overt attention.

Early reports from Buswell (1935) and Yarbus (1967) (see DeAngelus and Pelz, 2009; Tatler et al., 2010 for modern replications) suggested a similar role of task on eye movement control during picture viewing. As is the case in real-world tasks, task-directed eye movements in pictures were observed in both studies while participants engaged in a variety of scene viewing tasks. For example, when Yarbus’ participant was asked to remember the positions of people and objects in a painting, they distributed their eye movements throughout the scene. Given instructions to estimate the ages of the people in the scene, however, the same participant viewing the same painting looked almost exclusively at the faces of the people in the scene, showing a clear bias to move attention in service of the current task goals.

More recent evidence further supports this strong role of task in determining the placement of eye movements in scenes (Henderson et al., 2007, 2009; Einhäuser et al., 2008; Castelhano et al., 2009). For example, Castelhano et al. (2009) found task instruction influenced both the frequency with which participants fixated non-target objects in a given scene and the amount of time they lingered on those objects. Participants searching for an object in a scene were less likely to fixate non-target objects and spent less time fixating those objects than participants trying to memorize a scene. A goal of the present study was to determine whether these task-driven differences in where participants move their eyes persist when neither task asks participants to look for a particular object.

In addition to affecting where we look in a scene, task also influences more quantitative aspects of eye movement behavior. Castelhano et al. (2009) found task-dependent differences in aggregate eye movement measures during a scene memorization task and a search task (e.g., scan path length, total number of fixations, and percent of scene area fixated) and in the amplitude of the first five saccades in a scene. They did not find differences in fixation duration (both mean and across the first five fixations) nor in mean saccade amplitude. In contrast, Mills et al. (2011) did find evidence for task-dependent changes in mean fixation duration and mean saccade amplitude when participants engaged in memorization, aesthetic judgment, free-viewing, and search tasks. Mills et al. (2011) also found task-dependent differences in the rate of change in fixation duration over the course of a trial (see also, Antes, 1974; Friedman and Liebelt, 1981; Unema et al., 2005; Nuthmann et al., 2010; Nuthmann, 2017), while Castelhano et al. (2009) found no effect of task on ordinal fixation duration.

Several major differences between these studies may have contributed to their different findings. First, as Mills et al. (2011) point out, Castelhano et al. (2009) compared an experimenter-directed task (search for an experimenter-defined object in a scene) with a participant-directed task (memorize the scene), where the participants determined what scene regions were relevant to their task. Mills et al. (2011) compared four participant-directed tasks. Second, participants in Castelhano’s study completed both of the tasks in a counterbalanced order while Mills’ participants only participated in one of the four tasks examined. It is possible that the order in which participants did the tasks in Castelhano’s study influenced their behavior on the second task. It is also possible that the differences found by Mills et al. (2011) were participant-driven, rather than task-driven. Third, Mills et al. (2011) task ended after 5 s of viewing, while Castelhano’s study extended for 10 s. Finally, both studies had relatively small sample sizes (*N* = 20 for Castelhano et al., 2009, *N* = 12–14 for Mills et al., 2011).

The two major goals of the present study were to (1) establish baseline metrics of eye movement behaviors in photographs of scenes, and (2) to explore how task influences both where the eyes move in a scene and the quantitative features of those eye movements. To accomplish this, we used two participant-directed tasks: a scene memorization and an aesthetic judgment task. Participants viewed each scene in our study for 12 s, allowing us to compare our results to both Castelhano and Mills’ studies. Our participants completed both tasks in a counterbalanced order, and, because of our much larger sample size (*N* = 100), we were well-powered to look at task × order interactions and subject-level effects and control for both in subsequent analyses.

## Materials and Methods

### Participants

One hundred fourteen experimentally naive University of California, Davis undergraduates with normal or corrected-to-normal vision were recruited from the UC Davis undergraduate subject pool. They received course credit in exchange for their participation. Fourteen participants’ data were replaced due to poor eye tracking (25% or greater signal loss over all trials; Henderson and Hayes, 2017), leaving 100 participants’ data available for analysis.

### Apparatus and Stimuli

Eye movements were monitored with a tower-mounted EyeLink 1000 eye tracker (spatial resolution 0.01°rms) sampling the right eye at 1000 Hz (SR Research, 2010). Participants were seated 85 cm from a 21′′ CRT monitor. Participants’ head movements were limited by a chin and forehead rest. One hundred luminance-matched images of real world scenes were presented at their full resolution (1024 × 768 px), which filled the entire viewable area of the monitor (26.5 × 20 degrees of visual angle). The experimental stimuli were presented using the SR Research Experiment Builder software (SR Research, 2010).

Scenes were primarily drawn from online image searches. The 100 scenes were chosen to represent 100 unique scene categories. Half of the images were indoor scenes, half were outdoor. Most of the scenes included man-made structures, though a small subset were entirely natural scenes (8). We avoided choosing scenes with humans and legible text.

### Procedure

Participants viewed each of the 100 scenes for 12 s under one of two sets of task instructions. For 50 scenes they were told to memorize the images for a later memory test. Participants’ memory for these images was tested after they had completed both tasks. Images were drawn from both task conditions and were presented in a random order with 50 new images. Participants indicated whether they remembered each image on a 6-point scale (see Ramey et al., 2019 for a full description of this task). The data from this memory task are not presented here.

For the other 50 scenes, they were asked to assess the aesthetic qualities of the image and, after the 12 s viewing period, responded whether they liked, felt neutral about, or disliked the image. This response was recorded by a RESPONSEPixx Handheld button box (VPixx Technologies). Task instruction order was counterbalanced across subjects and scenes such that all subjects viewed all 100 images and each of the 100 images appeared equally under the two viewing task conditions across all subjects. A drift correction was performed prior to the onset of each scene. This resulted in all participants beginning their scene viewing at the center of each image.

### Data Analysis

#### Data Preparation

Eye movement data were imported into MATLAB using the EDFConverter tool. Participants’ eye tracking data was first assessed for missing data: any participant with track-loss of greater than 25% was removed from further analysis and replaced with a new subject. Participants who met this criteria were then assessed at the trial level: any trial in which participants’ eyes were tracked for less than 75% of the duration of the trial were also excluded from further analyses. This resulted in a loss of 1.2% of experimental trials.

To be consistent with previous work (Castelhano et al., 2009; Mills et al., 2011), we trimmed fixation durations that were extremely short (50 ms or shorter) or very long (1500 ms or longer) from our analyses. This trim resulted in a loss of 2.3% of fixations.

#### Object Segmentations

**FIGURE 1:**
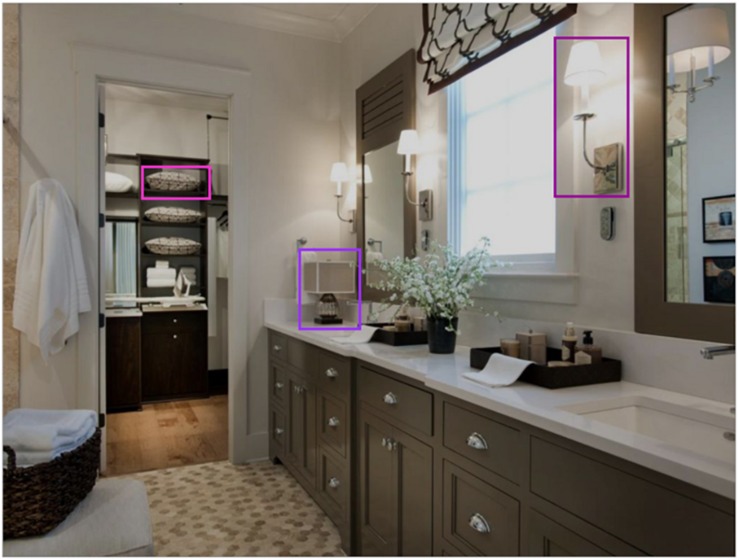
Example scene with three objects selected for analysis We used these objects to examine task-driven differences in eye movement behaviors to discrete objects.

In order to compare our results to those of Castelhano et al. (2009), we replicated their analysis of eye movement behavior in relation to objects. All objects in our scenes were labeled using LabelMe, an online annotation tool (Russell et al., 2008). We placed rectangular bounding boxes around three objects chosen randomly from the list of objects within each scene with the following limits: (1) the object was not occluded by any other object, (2) objects were of similar size, and (3) the bounding boxes for the three objects in a scene should not touch or overlap. Any fixations falling within the rectangular regions of interest around those objects were considered fixations on the object. Likewise any saccades made to these regions were considered eye movements to the object (Figure 1).

#### Analyses

To examine the differences between eye movement metrics between our two task conditions, we employed linear mixed effects models (LMEs) with task (memorization or aesthetic judgment) and task order as fixed effects and subject and scene as crossed, random intercepts. These analyses were completed using the package *lme4* (version 1.1-21, Bates et al., 2015) in *R* (R Core Team, 2019). Object-level analyses were completed in Python^1^.

## Results

### Broad Metrics

In the interest of establishing baseline metrics of eye movement behaviors in scenes, we first examined our participants’ behavior without regard to their viewing task. On average, participants made fixations that lasted 298 ms (SD = 64 ms). They made 32.83 fixations (SD = 7.10) per 12 s trial, or 2.74 fixations per second. When participants moved their eyes, they moved on average 4.58° per saccade (SD = 1.17°). Across the entire 12 s trial, they moved their eyes on average 147.40° (SD = 46.02°), or approximately 12° per second.

The amount of time participants remained at the center of the screen after the scene appeared (the initial saccade latency) tended to be shorter on average (*M* = 285 ms, SD = 119 ms) than the average fixation duration (*M* = 298 ms, SD = 64). The initial eye movement in a scene also tended to be shorter than the average eye movement by about 1° (*M* = 3.73, SD = 2.30). These results are summarized in Table 1.

**TABLE 1:** Mean and standard deviation of eye movement measures in a 12 s trial.

	**Mean**	**SD**
Fixation duration	298 ms	64 ms
Number of fixations	32.83	7.10
Saccade amplitude	4.58°	1.17°
Initial saccade latency	285 ms	119 ms
Initial saccade amplitude	3.73°	2.30°
Scan path length	147.40°	46.02°

Participants’ mean fixation duration and saccade amplitude was not constant over the course of a trial. With the exception of the initial saccade latency and amplitude, fixations made earlier in the trial tended to last less time on average than fixations made later in the trial (first five fixations mean = 270 ms, SD = 47 ms; last five fixations mean = 288 ms, SD = 39 ms), while early saccades tended to be longer than later saccades (first five saccades mean = 4.94°, SD = 1.10°; last five saccades mean = 3.48°, SD = 1.22°).

Previous work has estimated the average eye movement in scenes to be 2–4° in length (Henderson and Hollingworth, 1998) and the average fixation duration to be 200–300 ms (Rayner, 2009). Here, we find a longer average eye movement (4.58°), with even longer saccades earlier in the trial, and average fixation durations nearer the top end of previous estimates (298 ms).

### Task-Dependent Differences

Participants in our experiment were engaged in two tasks: a memorization task in which they were asked to memorize the scenes for a later test, and an aesthetic judgment task in which they were told to judge the pleasantness of each scene. We sought to determine whether these two different task instructions changed participants’ eye movement behavior. All participants completed both tasks in a counterbalanced order, so we first examined whether the order in which participants completed the two tasks influenced their eye movement patterns. We found task order significantly interacted with almost all of our eye movement measures of interest. Because we were interested in whether task instruction influenced eye movements (and not whether it interacts with fatigue or some other factor that may arise in the second task block), we limited further analyses to data from participants’ first task block only. Thus, the analyses reported below are between-subjects and have an *N* = 50 in each task condition^2^.

#### Mean Differences

To assess the differences in participants’ eye movement behaviors in our two task conditions, we compared mean fixation duration, mean saccade amplitude, total fixation number, initial saccade latency (e.g., the duration between the onset of the scene and the first saccade), total scan path length (e.g., the summed distance between fixations), and the spread of fixations through the scene. We used two measures of fixation spread: percent of the scene fixated (the summed area within a 2° window around every fixation divided by the total scene area; Castelhano et al., 2009) and the standard deviation of the *x*- and *y*-coordinates from the center of the scene (“dispersion from center,” Anliker, 1976).

For each dependent variable of interest, we fit a LME to trial means treating task condition as a fixed effect and participant and scene as random effects. We found no significant differences across the two task conditions in any of the dependent variables of interest, suggesting that, at the mean-level, participants’ eye movement behaviors in the memory and aesthetic judgment tasks were very similar. The results of all LMEs are reported in Table 2.

**TABLE 2:** Descriptive statistics and LME results for task-driven differences in eye movement measures.

	**Memorization**		**Aesthetic Judgment**		***LME***		
	**Mean**	**SE**	**Mean**	**SE**	**χ^2^**	***df***	***p***
Fixation duration (ms)	304	10	292	8	2.02	1	0.16
Saccade amplitude (°)	4.66	0.18	4.63	0.15	0.03	1	0.86
Number of fixations	32.72	1.10	33.72	0.91	1.50	1	0.22
Init. saccade latency (ms)	295	19	282	15	2.07	1	0.15
Scan path length (°)	147.62	6.83	153.37	6.37	1.00	1	0.32
Percent scene fixated (%)	12.52	0.46	13.10	0.42	2.35	1	0.13
Disp. from center (pixels)	297.76	5.72	291.33	4.80	1.62	1	0.20

#### Temporal Differences

While previous work has similarly found no task-driven differences in fixation duration and saccade amplitude at the mean-level, there is evidence that task influences how these variables change over the course of a trial. Castelhano et al. (2009) found task-driven saccade amplitude differences across the first five fixations in a trial, while Mills et al. (2011) found task-driven differences in fixation duration in the first 1 and 2 s of their aesthetic judgment and memorization task trials.

**FIGURE 2:**
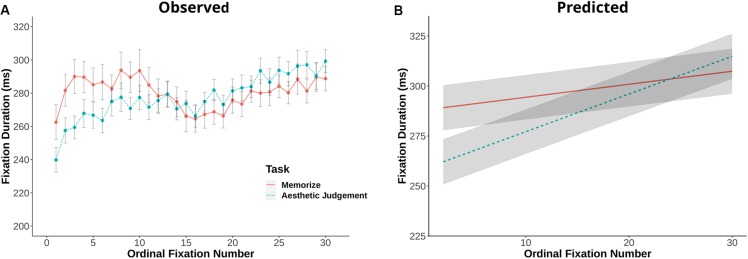
**(A)** Fixation duration plotted against ordinal fixation number for the memorize and aesthetic judgment conditions. Dots indicate mean values, error bars plot the 95% confidence interval. **(B)** LME predicted values of fixation duration by ordinal fixation number for the two task conditions.

**FIGURE 3:**
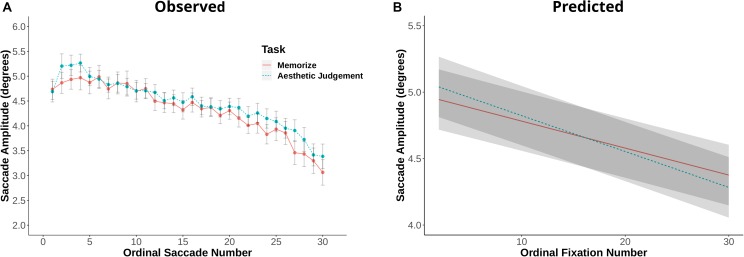
**(A)** Saccade amplitude plotted against ordinal fixation number for the memorize and aesthetic judgement conditions. Dots indicate mean values, error bars plot the 95% confidence interval. **(B)** LME predicted values of saccade amplitude by ordinal fixation number for the two task conditions.

Fixation duration and saccade amplitude are plotted by ordinal fixation number in Figures 2, 3, respectively. To assess the possibility of temporal effects of task in our data set, we first examined how saccade amplitude and fixation duration changed over the course of a trial in our memorization and aesthetic judgment tasks. Because subjects varied in the number of fixations they made on a given trial, this analysis was limited to the first 30 fixations/saccades in a trial. LMEs with fixation number and task condition as fixed effects and subject and scene as random effects revealed significant interactions between task and fixation number for both fixation duration [χ^2^(1) = 143.26, *p* < 0.001] and saccade amplitude [χ^2^(1) = 7.37, *p* = 0.006]. The main effect of task was not significant for fixation duration [χ^2^(1) = 1.73, *p* = 0.19] or saccade amplitude [χ^2^(1) = 0.00, *p* = 0.98]. The interactions suggests that the two tasks lead to different patterns of effects across ordinal fixation number: early fixation durations were longer in the Memorization task than in the aesthetic judgment task, while later fixation durations were more similar (Figure 2). Meanwhile, early eye movements in the memorization task tended to be shorter and later eye movements longer compared to the aesthetic judgment task (Figure 3). Thus, unlike previous work, we find task-driven differences in both saccade amplitude and fixation duration over the course of our 12 s trial.

In the interest of directly comparing the results of the present study to those of Castelhano et al. (2009), we repeated the same analyses including only the first- and last-five fixations per trial. In our study, a linear mixed effects model with fixation number and task as fixed effects and scene and subject as random effects revealed a significant effect of task on early fixation durations: participants’ first five fixations lasted longer in the memorization condition than in the aesthetic judgment condition [χ^2^(1) = 11.44, *p* < 0.001]. We did not find a significant effect of task on the duration of the last five fixations in a trial [χ^2^(1) = 0.75, *p* = 0.386], nor on the first and last five saccade amplitudes [first five: χ^2^(1) = 1.15, *p* = 0.284; last five: χ^2^(1) = 0.03, *p* = 0.856]. However, there was a task by fixation number interaction for the first five saccade amplitudes, [χ^2^(1) = 4.32, *p* = 0.038]. These results contrast with Castelhano et al. (2009), who found no difference in fixation durations on early trial fixations, but did find an effect of task on early trial saccade amplitudes.

Castelhano et al. (2009) compared eye movement behaviors during a visual search task and a memorization task. Mills et al. (2011), on the other hand, compared four task conditions, two of which were the same tasks used in the present study: a memorization task and an aesthetic judgment task. Mills et al. (2011) found fixation duration differences between these two tasks at 1 and 2 s time points, but not over the full length of their 5 s trial. They found no difference in saccade amplitudes. To ease comparison between our two studies, we conducted a similar analysis to theirs by looking at 1, 2, and 5 s time points in our data. LMEs with task as a fixed effect and subject and image as random effects revealed significant differences in fixation duration at all three time points in our data [1 s: χ^2^(1) = 5.71, *p* = 0.016; 2 s: χ^2^(1) = 13.59, *p* < 0.001; 5 s: χ^2^(1) = 7.92, *p* = 0.005]. Similar to Mills et al. (2011), we did not find any differences in saccade amplitude across those same time points [1 s: χ^2^(1) = 0.53, *p* = 0.46; 2 s: χ^2^(1) = 1.31, *p* = 0.253; 5 s: χ^2^(1) = 0.06, *p* = 0.81]. These results largely overlap with Mills et al. (2011) findings, with the exception of the 5 s time point: we found differences in fixation duration at 5 s, while Mills et al. (2011) did not.

#### Distribution-Level Differences

Another way to examine the differences in participants’ behaviors across our two task conditions is to compare the full distributions of the variables of interest with a shift function (Doksum, 1974, 1977; Doksum and Sievers, 1976; Wilcox, 1995; Rousselet et al., 2017). Shift functions quantify the differences between two distributions by dividing each distribution into quantiles, subtracting one distribution’s quantile boundary from the corresponding quantile boundary of the other, then plotting the differences against the quantiles of the first distribution. In this way, shift functions can reveal differences in both the position and spread of the two distributions. For example, if one distribution has more data in its tail than the other, its later quantile means will be greater than the corresponding quantile means for the other distribution. Using this sort of distribution-level comparison in the present study provides a more complete understanding of how our two task conditions influenced participants’ eye movement behaviors than an analysis of the means alone.

**FIGURE 4:**
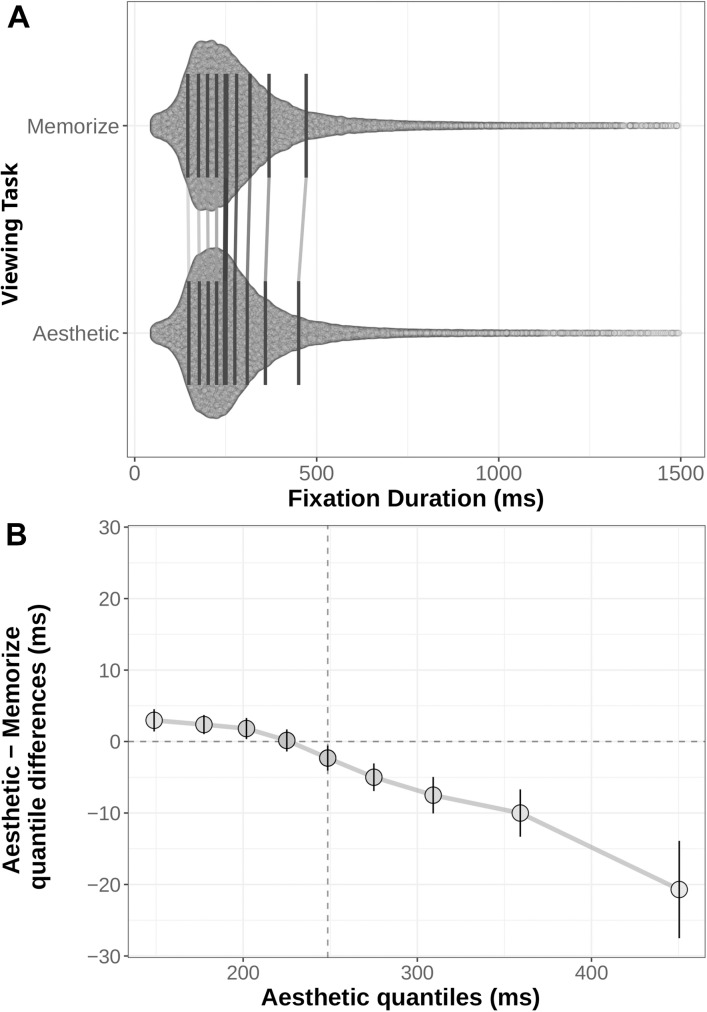
**(A)** Distribution of fixation durations under Memorization and Aesthetic Judgment task conditions. Black lines indicate the nine quantile means for each distribution. Gray lines connect corresponding quantile means. **(B)** Shift function comparing the memorization and aesthetic judgement conditions. Circles mark the difference estimates for each decile. Error bars are 95% bootstrap confidence intervals. The horizontal dotted line denotes no difference between conditions: quantile difference estimates whose error bars cross this line are not reliably different. The vertical dotted line marks the median of the aesthetic distribution (i.e., the 5th decile).

**FIGURE 5:**
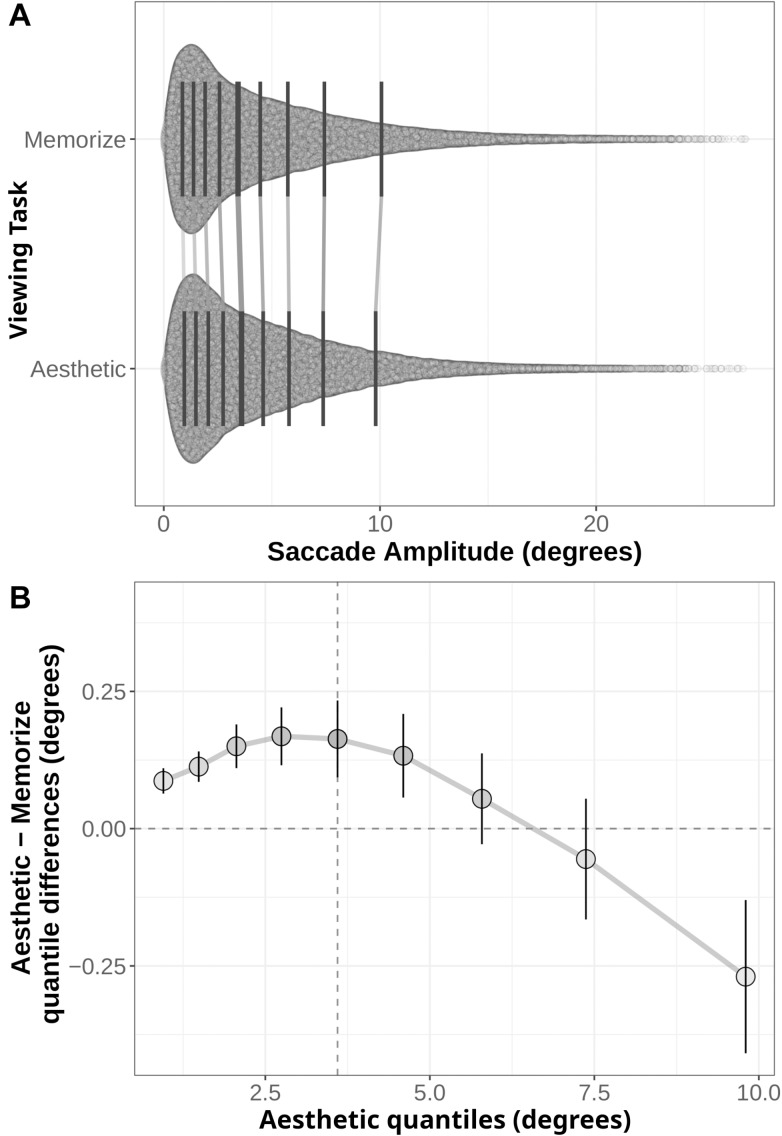
**(A)** Distribution of saccade amplitudes under the Memorization and Aesthetic Judgment task conditions. Black lines indicate the nine quantile means for each distribution. Gray lines connect corresponding quantile means. **(B)** Shift function comparing the memorization and aesthetic judgement conditions. Circles mark the difference estimates for each decile. Error bars are 95% bootstrap confidence intervals. The horizontal dotted line denotes no difference between conditions: quantile difference estimates whose error bars cross this line are not.

We generated shift functions for the fixation duration and saccade amplitude distributions from our two task conditions using the *R* package *rogme* (Rousselet et al., 2017*).* The distributions were first binned into deciles (Figures 4A, 5A). The deciles for the memorization condition were then subtracted from the corresponding aesthetic judgment condition deciles. The resulting difference scores are plotted with their 95% bootstrap confidence intervals against the aesthetic judgment deciles. The resulting function quantifies where the two conditions distributions differ from each other: non-zero difference estimates indicate differences between the two distributions in that quantile. Estimates whose bootstrapped confidence intervals do not overlap with zero can be considered reliable or significant differences between the two distributions.

The distributions and shift function for fixation duration across our two task conditions are plotted in Figure 4 and the decile estimates and bootstrapped confidence intervals are listed in Table 3. The early decile estimates for the memorization and aesthetic judgment distributions fall within a few milliseconds of each other, suggesting the bulk of the distributions were overlapping. However, the overall negative trend indicates a difference between the tails of the two fixation duration distributions. Participants with instructions to memorize a scene made more long-duration fixations than participants with instructions to judge the aesthetic qualities of the scene.

**TABLE 3:** Quantile difference estimates between task condition distributions.

	**Fixation duration (ms)**			**Saccade amplitude**		
**Decile**	**Difference estimate**	**95% CI lower**	**95% CI upper**	**Difference estimate**	**95% CI lower**	**95% CI upper**
1	**2.97**	**1.41**	**4.53**	**0.09°**	**0.06**	**0.11**
2	**2.38**	**1.03**	**3.72**	**0.11°**	**0.09**	**0.14**
3	**1.82**	**0.34**	**3.30**	**0.15°**	**0.11**	**0.19**
4	0.16	−1.39	1.70	**0.17°**	**0.12**	**0.22**
5	−**2.31**	−**4.07**	−**0.55**	**0.16°**	**0.09**	**0.23**
6	−**4.99**	−**6.93**	−**3.05**	**0.13°**	**0.06**	**0.21**
7	−**7.50**	−**10.06**	−**4.95**	0.06**°**	−0.03	0.14
8	−**10.01**	−**13.32**	−**6.69**	−0.06**°**	−0.17	0.05
9	−**20.71**	−**27.52**	−**13.89**	−**0.27°**	−**0.41**	−**0.13**

*Bold values indicate reliable differences between the distributions.*

The distributions and shift function for saccade amplitudes are plotted in Figure 5. In this case, we find differences across the entire shift function. The positive difference values over the first half of the function indicate the distribution of saccade amplitudes given aesthetic judgment instructions is shifted to the right relative to the memorization distribution. However, the negative difference value in the last quantile indicates the memorization distribution has more observations in the tail than the aesthetic judgment distribution. Taken as a whole, these quantile differences indicate participants in the memorization condition tended to make more short-amplitude saccades than participants in the aesthetic judgment condition, but were also more likely to make very long saccades (as indicated by the 0.27° difference in the last quantile bin).

In sum, while we did not find a task-dependent difference at the level of the mean, task instruction did influence both fixation duration and saccade amplitude over the course of a trial (as indicated by the temporal analyses described above) and at the level of the distribution (as indicated by the distributional shift analyses).

### Where the Eyes Moved

Task instructions can influence both *when* the eyes move and *where* the eyes move in a scene. Up to this point, we have explored task-driven timing differences in global fixation duration and saccade amplitude. Now, we will explore whether our two tasks drove participants to look differently at the level of local objects. To do so, we utilized the same method described in Castelhano et al. (2009): three objects were randomly selected in each scene. A rectangular interest area was drawn around each of the three objects. Any fixations within or saccades to the interest area surrounding those three objects were included in this analysis (see Figure 1). Differences between the two task conditions were tested by independent-sample *t*-tests. We used Bonferroni-adjusted *p*-values to correct for family-wise error. The results of these tests are reported along with descriptive statistics in Table 4.

**TABLE 4:** Task-driven differences in objects fixated.

	**Memorization**		**Aesthetic judgment**				
	**Mean**	**SE**	**Mean**	**SE**	***t***	***df***	***P***
Proportion of objects fixated	0.48	0.02	0.5	0.01	1.05	98	1.000
Avg. saccade amplitude to object (**°**)	5.32	0.17	5.31	0.16	0.03	98	1.000
Avg. fixation duration (ms)	260	9	246	6	0.51	98	1.000
Avg. first fixation duration (ms)	253	9	235	5	0.47	98	1.000
First gaze duration (ms)	325	11	291	6	1.98	98	0.41
First gaze fixation count	1.05	0.02	1.04	0.02	0.51	98	1.000
Total time (ms)	1116	37	1066	27	0.28	98	1.000
Total number of fixations	3.59	0.11	3.67	0.10	0.52	98	1.000

There were no significant, task-driven differences in any of the measures investigated (see Table 4). These results contrast with Castelhano et al. (2009) comparison of memorization and search. They found task-driven differences in the first gaze duration, proportion of objects fixated, the number of fixations during the first gaze at an object, the total time spent fixating the objects, and the total number of fixations on the objects across their two tasks (memorization and search). In our case, subjects fixated about half of the objects selected for this analysis in both conditions. Castelhano et al. (2009) reported 66% of the objects fixated for the memorization condition and 53% for their search condition. Fixation durations on objects during the memorization condition lasted 260 ms on average (SD = 89 ms), while fixation durations on objects during the aesthetic judgment task tended to be shorter (*M* = 246 ms, SD = 6 ms). Fixations on objects in Castelhano et al. (2009) study tended to be longer than those reported here (memorization: 290 ms, search: 279 ms). The average gaze duration on the objects in our study was 325 ms in the memorization condition (SD = 107 ms) and 291 ms in the aesthetic judgment condition (SD = 65 ms). These values lie closer to the search task gaze durations from Castelhano et al. (2009) study (348 ms) than to their memorization task (439 ms). Participants fixated the objects chosen about 3.5 times, spending a total of about 1 s fixating them over a 12 s trial.

## Discussion

In this study, we found no evidence that participants’ fixation durations and saccade amplitudes varied with task at the mean level. However, we found task-driven differences in fixation duration and saccade amplitude over the course of a trial and at the level of the distribution. Castelhano et al. (2009) found no evidence that fixation duration and saccade amplitude were influenced by task instruction when comparing scene memorization and visual search, with the exception of a task-driven difference in early trial saccade amplitudes. Mills et al. (2011) similarly found few differences between an aesthetic judgment and memorization task: participants exhibited significantly different fixation durations in the first 2 s of a trial, but not across the entire 5 s trial period. Mills et al. (2011) found no task-driven difference in saccade amplitudes between their memorization and aesthetic judgment task.

Unlike Castelhano et al. (2009) comparison of memorization and search, we found no evidence for task-driven differences in our aggregate trial data when comparing memorization and aesthetic judgment. Participants’ scan path lengths, dispersion of fixations, initial saccade latencies, and total number of fixations were similar in both of our tasks and we found no difference in *where* our participants directed their gaze. Our temporal results also differed from those of Castelhano et al. (2009). We found significant effects of task on ordinal fixation duration and saccade amplitude. In addition, a replication of their temporal analyses revealed a significant effect of task on early trial fixations, but not on late-trial fixations or early- or late-trial saccade amplitudes. Our results aligned more closely with Mills et al. (2011): we found similar temporal effects of task on fixation duration and saccade amplitudes. While Mills found effects of task on fixation durations at 1 and 2 s time points, but not across their full 5 s trial, we found an effect of task throughout the first 5 s of our 12 s trials. We, like Mills et al. (2011), found no effect of task on saccade amplitudes over the first 1, 2, and 5 s of our trials.

The most likely reason our findings differ from Castelhano et al. (2009) is the difference in task demands in our studies. Castelhano et al. (2009) tasks consisted of a participant-directed memorization task and an experimenter-directed search task, wherein participants looked for an experimenter-specified object within a scene. The task demands in these two tasks likely differ more than those of the memorization and aesthetic judgment tasks used in the present study, which are both participant-directed tasks (Mills et al., 2011). Furthermore, search tasks, whether for an object within a scene [as in Castelhano et al. (2009)] or for a small, embedded letter [as in Mills et al. (2011)], seem to have a particularly strong influence on eye movement behavior in scenes. Fixations tend to be more brief (Castelhano et al., 2009; Mills et al., 2011; Nuthmann, 2017) and saccade amplitudes shorter (Castelhano et al., 2009; Mills et al., 2011) in search tasks compared to other tasks. These effects are likely driven by strong attentional guidance to potential target locations during search tasks (e.g., Wolfe, 1994; Zelinsky, 2008; Malcolm and Henderson, 2010; Võ and Henderson, 2011; Wolfe et al., 2011a) and, because trials are typically time-limited, the explicit nature of a search task encourages participants to move their eyes quickly until the target is found.

We did not find evidence that participants changed *where* they moved their eyes under our two task conditions. These results also contrast with Castelhano et al. (2009) findings: their task demands significantly influenced a variety of measures of attention to objects within their scenes, with participants in the memorization condition fixating more objects and for longer than participants in the search condition. This contrast between the results of the present study and Castelhano et al. (2009) is, again, likely driven by the unique demands of the search task in Castelhano et al. (2009) study. One might expect participants with instructions to memorize a scene would spend more time fixating objects than participants in the aesthetic judgment condition, but we did not find evidence that this was the case. Instead, the aesthetic judgment and memorization tasks seemed to drive subjects’ attention through the scene in a similar manner.

While the task-driven differences found in the present study are not as apparent as those reported by Castelhano et al. (2009), the temporal and distribution-level effects suggest that even in tasks with similar, participant-directed task demands, participants adjust their eye movement behaviors to meet those demands. During early scene-viewing, a memorization task drove fixation durations up compared to an aesthetic judgment task. This is also evidenced at the distribution-level: participants in the memorization task made more long-duration fixations than participants in the aesthetic judgment condition. These differences in fixation duration may be evidence of subjects’ memorization strategy: spending more time at each fixation may allow for better subsequent memory for the fixated regions (Hollingworth and Henderson, 2002). Participants in the memorization condition were also more likely to make shorter amplitude saccades than participants in the aesthetic judgment task (as revealed by the distribution-level analysis, Figure 5). This effect may also be indicative of subjects’ strategy during the respective tasks: participants in the memorization task may have more thoroughly explored local scene regions in an effort to better remember fine scene detail.

An alternative explanation for the differences in behavior across our task conditions is that participants may have been less engaged in one of the experimental tasks. Participants were immediately probed for their response in the aesthetic judgment condition, while in the memorization task participants were not tested on their memory until the end of the task. This may have led to participants being less engaged in the memorization task than in the aesthetic judgment task. Task engagement can affect eye movement behavior during scene viewing. For example, low engagement may lead to more mind wandering, which is known to affect gaze behavior during scene viewing (e.g., Krasich et al., 2018). While some markers of mind wandering map on to our memorization data (longer fixation durations), others do not (shorter saccades).

Much research, including models of overt attention, has focused on why the eyes move *where* they do within a scene. Recently, Nuthmann (2017) explored the effects of local feature information and task on fixation durations in scenes. She found significant relationships between low-level feature information at the current fixation location (luminance, contrast, edge density, clutter, and the number of segments) and fixation duration. These features are typical of those used to predict regions likely to draw overt attention in traditional saliency models (e.g., Itti and Koch, 2000). Nuthmann also found differences in how these local features influenced fixation duration across task. In conjunction with the present work, the results of Nuthmann’s (2017), Mills et al. (2011), and Castlehano and colleagues’ studies strongly suggest that it is just as important to understand why the eyes move *when* they do within a scene. Further, models of scene viewing behavior will be met with most success if viewing task is taken into consideration.

### Distribution-Level Analysis

A typical analysis in cognitive psychology research consists of comparing means to assess differences between conditions. While such analyses provide information about the central tendency of the distributions of interest, the experimental manipulation may affect any part of the distribution. For example, in this study, we found no differences in the mean fixation duration or mean saccade amplitude across our two tasks. However, a distribution-level analysis using a shift function (Doksum, 1974, 1977; Doksum and Sievers, 1976; Rousselet et al., 2017) revealed that participants in the memorization condition tended to make more short saccades and longer fixations than participants in the aesthetic judgment condition. The mean level analysis suggested that there was no effect of task, while the distribution-level analysis revealed relatively large, reliable differences between the tails of the fixation duration distributions and across the entire saccade amplitude distribution.

Recently, there has been an increase in popularity of visualizing differences between groups with distribution-level data (e.g., scatter plots, violin plots, raincloud plots, etc.) rather than simply plotting means and a measure of variability (e.g., bar plots, box and whisker plots, etc.). These plots of individual subject- or full distribution-level data provide much more information about how the experimental manipulations affected participants’ performance. Similarly, analyzing differences in the distributions of the experimental groups provides much more information than analyzing differences in their mean performance. Future work would benefit from applying methods like the shift function to their own data. For a thorough discussion of the shift function, please see Rousselet et al. (2017).

### Metrics of Eye Movements in Scenes

A goal of the present work was to set out baseline estimates of the properties of eye movements during scene viewing (Table 1). Having estimates of typical scene viewing behavior allow us to better assess how experimental manipulations change those behaviors. Further, because our data come from a “typical” sample (e.g., healthy college students), the estimates we provided here will have use for assessing how the eye movement behaviors of participants from other groups (e.g., children, older adults, patient-populations) compare to those of the typical sample group.

## Conclusion

Eye movement behaviors are influenced by a wide variety of internal and external forces. Here, we provide evidence that two tasks with similar demands can yield different patterns of eye movements. To arrive at this conclusion, we supplemented traditional mean-level analyses with temporal and distribution-level analyses. We posit the distribution-level analyses, such as the shift function used here, are an underutilized, powerful method of assessing differences between two conditions. Finally, we provide a baseline characterization of eye movement behaviors during real-world scene viewing. These baseline estimates can serve as a useful tool for future research.

## Data Availability Statement

The raw data supporting the conclusions of this article will be made available by the authors, without undue reservation, to any qualified researcher.

## Ethics Statement

The studies involving human participants were reviewed and approved by the IRB Administration Office of Research University of California, Davis. Written informed consent for participation was not required for this study in accordance with the national legislation and the institutional requirements.

## Author Contributions

JH and TH conceived and designed the study. JG contributed to the data collection. DC and EH conducted all analyses. DC wrote the manuscript.

## Conflict of Interest

The authors declare that the research was conducted in the absence of any commercial or financial relationships that could be construed as a potential conflict of interest.

## Footnotes

1 Python Software Foundation. *Python Language Reference, Version 2.7*. Available at: http://python.org (accessed September 15, 2018).

2 The within-subjects analyses revealed significant differences in subjects’ mean fixation duration, saccade amplitudes, scan path length, fixation number, and dispersion. However, despite being significant, the differences were extremely small (e.g., 5 ms difference between the two tasks’ mean fixation durations) and difficult to interpret due to the task × order interaction.
